# Interannual and seasonal asymmetries in Gulf Stream Ring Formations from 1980 to 2019

**DOI:** 10.1038/s41598-021-81827-y

**Published:** 2021-01-26

**Authors:** Adrienne Silver, Avijit Gangopadhyay, Glen Gawarkiewicz, E. Nishchitha S. Silva, Jenifer Clark

**Affiliations:** 1 School for Marine Science and Technology, University of Massachusetts, Dartmouth, MA 02747 USA; 2 Woods Hole Oceanographic Institution, Woods Hole, MA 02543 USA; 3 Boston University Earth and Environment, Boston, MA 02215 USA; 4 Jenifer Clark’s Gulfstream, 3160 Lacrosse Court, Dunkirk, MD 20754 USA

**Keywords:** Physical oceanography, Attribution

## Abstract

As the Gulf Stream separates from the coast, it sheds both Warm and Cold Core Rings between $75^\circ$ and $55^\circ \,\hbox {W}$. We present evidence that this ring formation behavior has been asymmetric over both interannual and seasonal time-scales. After a previously reported regime-shift in 2000, 15 more Warm Core Rings have been forming yearly compared to 1980–1999. In contrast, there have been no changes in the annual formation rate of the Cold Core Rings. This increase in Warm Core Ring production leads to an excess heat transfer of 0.10 PW to the Slope Sea, amounting to 7.7–12.4% of the total Gulf Stream heat transport, or 5.4–7.3% of the global oceanic heat budget at $30^\circ \,\hbox {N}$. Seasonally, more Cold Core Rings are produced in the winter and spring and more Warm Core Rings are produced in the summer and fall leading to more summertime heat transfer to the north of the Stream. The seasonal cycle of relative ring formation numbers is strongly correlated (r = 0.82) with that of the difference in upper layer temperatures between the Sargasso and Slope seas. This quantification motivates future efforts to understand the recent increasing influence of the Gulf Stream on the circulation and ecosystem in the western North Atlantic.

## Introduction

The Gulf Stream carries more than half of the total annual oceanic heat transported toward subpolar regions in the northern hemisphere^1–3^. Part of this heat is advected by both Warm and Cold Core Rings formed from the Gulf Stream meanders in the Slope and Sargasso Seas, respectively. Two recent studies by Gangopadhyay et al.^4,5^ have shown that there has been a significant regime shift in terms of Warm Core Rings formed in the Gulf Stream between 75$^{\circ }$ and 55$^{\circ }$ W. The average has increased by 15 Warm Core Rings per year—from 18 per year during 1980–1999 to an average of 33 per year in the 2000s, largely affecting the continental shelf and slope waters of the northeast US and Canada. Environmental regime shifts have been shown to have long-lasting effects on ecosystems^6^. Changes in the North Atlantic shelf ecosystem in recent years have been noted with increased warming events and shifts in fish recruitment^7,8^. Pershing et al.^9^ also stated that the Gulf of Maine in the last decade (2005–2015) has been warming faster than 99% of the world’s oceans.

Though Warm Core Rings are frequently studied due to their impact on the New England shelf, the Cold Core Rings have received relatively less attention after their extensive study in the early eighties^10^ except for a few recent studies^11–13^. The Gangopadhyay et al.^4,5^ studies raised a very important question regarding ring formation from the Gulf Stream—does the formation rate of Cold Core Rings to the south of the Gulf Stream exhibit similar temporal and geographic patterns to the formation of Warm Core Rings? Using a newly developed census for Cold Core Rings, we examine the temporal behavior of the formation rates for Cold Core Rings versus Warm Core Rings at both interannual and seasonal time-scales.

## Results

### Ring formation: 1980–2019

**Figure 1:**
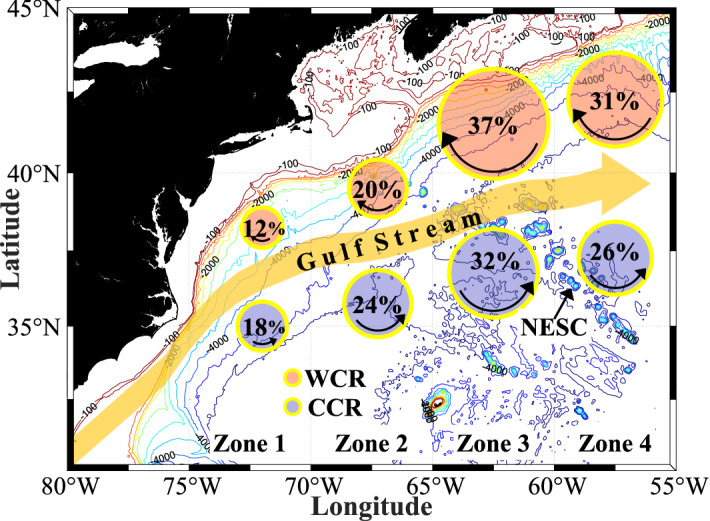
Geographic distribution of Warm and Cold Core Ring formation during 40 years of study (1980–2019). The four different zones within the $75^{\circ }$–$55^{\circ }\,\hbox {W}$ region are: Zone 1: $75^{\circ }$–$70^{\circ }\,\hbox {W}$; Zone 2: $70^{\circ }$–$65^{\circ }\,\hbox {W}$; Zone 3: $65^{\circ }$–$60^{\circ }\,\hbox {W}$; Zone 4: $60^{\circ }$–$55^{\circ }\,\hbox {W}$. A total of 1014 Warm Core Rings and 1023 Cold Core Rings were documented over the 40-year period. Percentages represent the number of Warm (Cold) Core Rings that are formed in that zone compared to total number of Warm (Cold) Core Rings. The map was generated using the MATLAB M_Map package^16^ with topography contours from ETOPO1^17^ and the coastline from Global Self-consistent, Hierarchical, High-resolution Geography Database (GSHHG)^18^.

The census for both Warm and Cold Core Ring formation, i.e. location and time, was developed following the methodology described later for the full domain ($75^{\circ }$–$55^{\circ }\,\hbox {W}$) and for four zones (Zone1: $75^{\circ }$–$70^{\circ }\,\hbox {W}$; Zone 2: $70^{\circ }$–$65^{\circ }\,\hbox {W}$; Zone 3: $65^{\circ }$–$60^{\circ }\,\hbox {W}$ and Zone 4: $60^{\circ }$–$55^{\circ }\,\hbox {W}$) (see Fig. 1).

During the 40-year study period, out of a total of 1014 Warm Core Rings formed, Zones 1 and 2 produced 116 and 204 respectively (12 and 20%). The more productive regions to the east, Zones 3 and 4 produced 376 and 318 rings respectively (37 and 31%). A geographically similar but weaker (more evenly distributed) pattern is seen in the Cold Core Rings. Out of the total of 1023 Cold Core Rings, 184 (18%) are formed in Zone 1, and 249 (24%) are formed in Zone 2. Much like the Warm Core Rings, Zone 3 is also most productive with 324 rings (32%) followed by 266 (26%) in Zone 4. The New England Seamount Chain (NESC) underlies the Gulf Stream in the northeastern part of Zone 3 and in the southwestern part of Zone 4, possibly enhancing the Warm and Cold Core Ring formation rate in Zones 3 and 4^14,15^ due to large-amplitude meandering of the Gulf Stream. Geographically the Gulf Stream behaves differently to the east and west of 60$^{\circ }$ W. Zones 1 and 2 produce more Cold Core Rings than Warm Core Rings with a total of 113 more Cold Core Rings produced than Warm Core Rings in these Zones over the whole study period. On the other hand, Zones 3 and 4 produce more Warm Core Rings than Cold Core Rings with a total of 104 more Warm Core Rings produced than Cold Core Rings over the whole study period.

#### Interannual asymmetry

**Figure 2:**
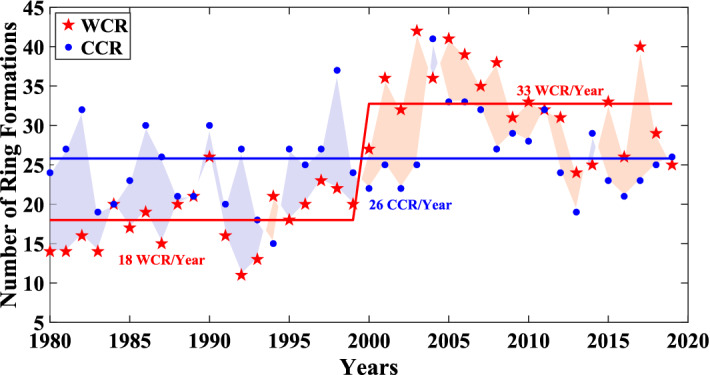
Interannual variability of the Warm Core Ring and Cold Core Ring formation between 1980 and 2019. The regime shift (denoted by the split in the red solid line) for the Warm Core Rings is significant at the turn of the century. Note the phase change from “More Cold Core Rings—Fewer Warm Core Rings” to “More Warm Core Rings—Fewer Cold Core Rings” after the year 2000. The average formation rates for different time-periods are indicated within the figure.

The interannual variability (IAV) shows (Fig. 2) large variation in the number of Cold Core Rings through the years but with no overall trend. This is in contrast to the previously reported IAV of Warm Core Rings with significant regime shift and trend over most of the 40-year period of study^4,5^. The Gulf Stream produced the most Cold Core Rings in 2004 (41) and the least in 1994 (15). The Cold Core Ring formation time-series was tested for detecting any regime shift using the method of Rodionov^19^ and two other methods following those used by Gangopadhyay et al.^4^ for the Warm Core Ring time-series (more detail in “Methods” section). No significant regime shift was detected in the Cold Core Ring formation series during the 40-year study period. Additionally, Fig. 2 shows that the Gulf Stream seems to have undergone a curious shift in the behavior of ring formation—from consistently generating more Cold Core Rings before 2000 (except in 1994) to consistently generating more Warm Core Rings after 2000 (except in 2004 and in 2014).

The annual number of Cold Core Rings produced by the Stream matches with what has been noted by previous studies^20^. Auer^20^ also noted a tendency of the Stream to produce more Cold Core Rings than Warm Core Rings during their period of study (1980s). This tendency switches after 2000 with the Stream producing more Warm Core Rings than Cold. Such regime shifts around 2000 were also detected in the time-series for the difference of rings (WCR − CCR) and for the total number of rings (WCR + CCR).

#### Seasonal asymmetry

**Figure 3:**
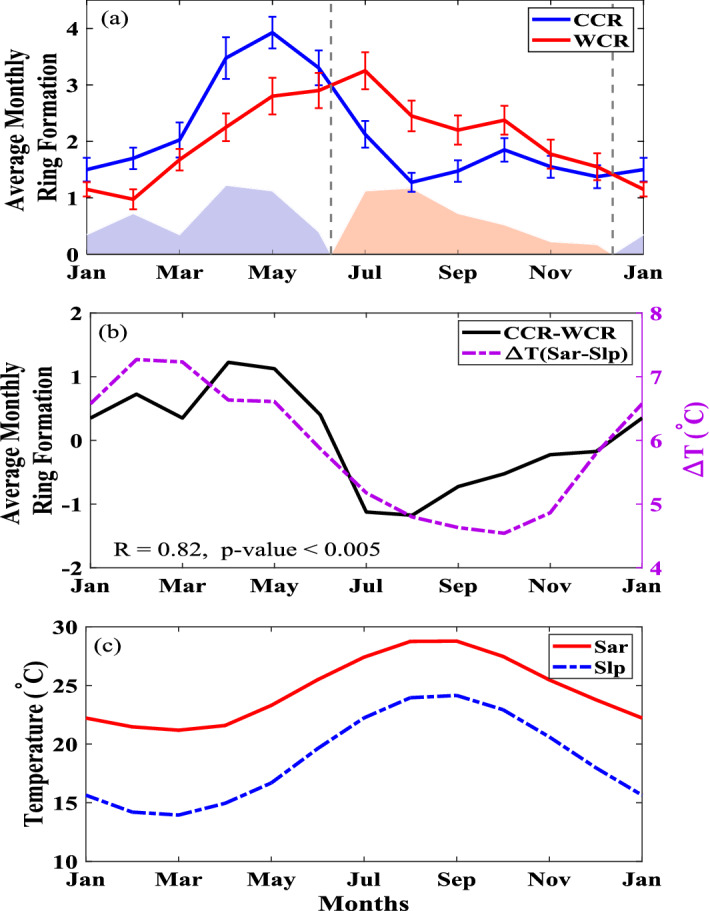
(**a**) Average Seasonal Cycles of Warm and Cold Core Ring formation between $75^{\circ }\,\hbox {W}$ and $55^{\circ }\,\hbox {W}$ from 1980 to 2019, error bars represent standard error from the mean. Shaded regions under the curve represent the difference between Warm and Cold Cord Ring rates of formation, emphasizing the shift from more Cold Core Rings forming during January through June to more Warm Core Rings forming during July through December. (**b**) The difference in ring formation numbers of Warm and Cold Core Rings (black line) and the monthly temperature difference (purple dashed line) between Sargasso and Slope waters from their respective monthly climatology shown in the bottom (**c**).

Two distinctly different seasonal patterns are evident for the Warm and Cold Core Ring formations (Fig. 3a). Cold Core Ring formation peaks in May, while the Warm Core Ring formation peaks in July. This phase shift in seasonality leads to more Cold Core Rings than Warm Core Rings being formed in the winter and spring, and more Warm Core Rings than Cold Core Rings being formed in the summer and fall. The switches in seasonal ring formation preference occur in June and December. This seasonality of the difference between the Warm and Cold Core Rings (CCR − WCR) is presented in Fig. 3b (black solid line) in a standardized (mean-zero) form.

A comparison of the relative formation numbers of Warm versus Cold Core Rings with the temperature difference between the Slope and Sargasso waters ($\Delta T$) shows a strong correlation with $R = 0.82; \, (p<< 0.01)$ (Fig. 3b). The value of $\Delta T$ reaches its minimum in July–November matching with the maximum amount of Warm Core Ring Formations. Additionally the maximum in $\Delta T$ occurs in spring (March–April) matching the relative peak in Cold Core Ring formation.

### Differential heat transport

The slope water receives heat in two ways: (i) by addition of Warm Core Rings which carry warm Sargasso water in their cores to the Slope, and (ii) by removing the Cold Core Rings which carry the colder slope water out of the Slope into the warmer Sargasso Sea. Thus, we expect the northward heat flux to increase if ring formation increases regardless of the ring’s sign. The regime shift in Warm Core Rings, doubling the number of rings per year, then equates to a net increase in the northward heat transport.

Given the average formation rate in Warm and Cold Core Rings per year, and the known average size of Warm/Cold Core Rings, an estimate of the increased heat transported onto the slope from the Gulf Stream can be calculated using the following equation

1 $$\begin{aligned} \Delta H = N_W(\pi r^2h)\rho C_p\Delta T_{W}-N_C(\pi r^2h)\rho C_p\Delta T_{C} \end{aligned}$$

**Figure 4:**
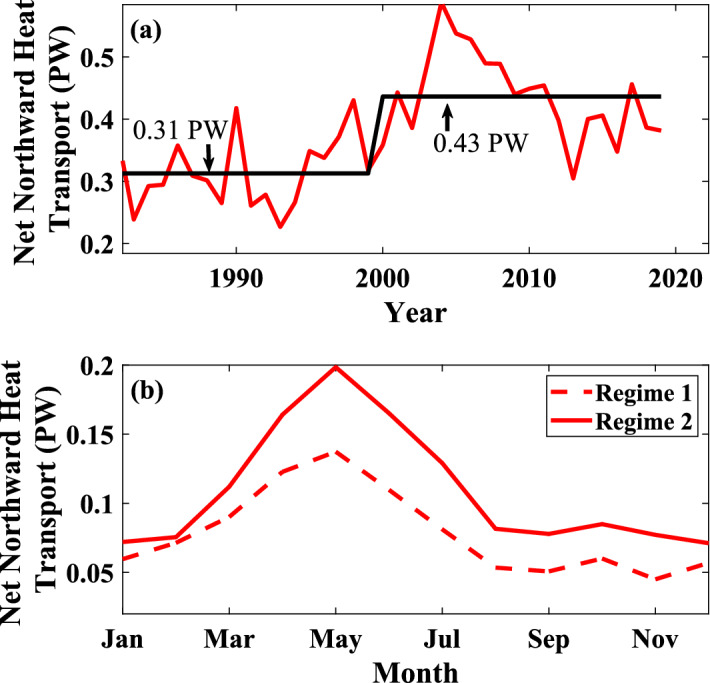
Net northward heat transport from Warm and Cold Core rings (**a**) shows the internal variability, (**b**) shows the seasonal variability, for before and after the regime shift in 2000.

Here, $N_W$ and $N_C$ are the numbers of Warm and Cold Core ring formations respectively, *r* is the average radius of a Warm or Cold Core Ring (80 km), *h* is the average depth of the Warm or Cold Core Ring core (500 m), $\rho$ is the density of sea water ($1025 \,\text{{kg\, m}}^{-3}$), $C_p$ is the specific heat of sea water (3850 J/kg $^{\circ }$C), and $\Delta T$ is the difference in water temperature between a Warm or a Cold Core Ring and the surrounding waters. The value of $\Delta T$ was estimated from the monthly temperature difference between Sargasso and slope waters taken from monthly climatology^21^ with Warm Core Rings having a positive $\Delta T_W$ and Cold Core Rings having a negative $\Delta T_C$ ($\Delta T$ is shown in Fig. 3b). The formation month of the Warm or the Cold Core Ring determines the $\Delta T$ used in the equation. The resulting Net Northward Heat Transport ($\Delta H$) is in Joules, which is an annual estimate, is then converted to Watts to compare with the global and Gulf Stream heat transports. This calculation was then done for each year within the study period (1980–2019). This interannual variability also shows a significant regime shift around the year 2000 in northward heat transport and is shown in Fig. 4a.

The year with the largest northward heat transport was 2004 for a total of 0.588 PW. The year with the lowest northward heat transport was 1993 with 0.227 PW, showing a large range in potential northward heat transport. Given that the total heat transport^1,22^ of the Gulf Stream, which is the majority of the heat transport in the North Atlantic, is 1.3 ± 0.3 PW, this 0.588 PW heat transport in 2004 is equivalent to 37–59% of the total Gulf Steam heat transport.

Similar to the interannual heat transport computation, the seasonal cycle of heat transport by the rings was obtained (Fig. 4b) using the monthly Warm and Cold Core Ring numbers. The monthly heat transport peaks in May with a total of 0.168 PW, which is equivalent to around 45% of the total annual heat transport from Gulf Stream rings.

When comparing the seasonal cycles of the heat transport before and after the regime shift (Fig. 4b), we find that the pattern remains almost identical, a large peak in May with a much smaller peak in October, but the magnitude of the peaks has increased. On average, the monthly northward heat transport increased by 0.031 PW from regime 1 to regime 2 which is about 33% of the average monthly heat transport for the total period (0.094 PW). The largest increases were seen in April through July with an increase on average of 0.052 PW per month and a max of 0.061 PW in May; whereas among the winter months, February saw the smallest increase of 0.004 PW.

## Discussion

Comparing the heat transport in regime 1 (before 2000) to regime 2 (after 2000) (Fig. 4a), it appears that the eddies transported an average of 0.124 PW more heat (annually) during regime 2 than in regime 1. Relative to the total GS heat transport, this excess is equivalent to 7.7–12.4% of the total GS transport. The global heat transport^2^ between $24^{\circ }$ and $36^{\circ }\,\hbox {N}$ is around $2.0 \pm 0.3$ PW. This indicates that the 0.124 PW excess heat on the Slope due to the increase in Warm Core Rings represents about 5.4–7.3% of the poleward global oceanic heat transport in the Northern Hemisphere. Seasonally the average monthly northward heat transport increased by about 33% from regime 1 to regime 2.

Increased numbers of Warm Core Rings not only increase the heat transported northward but could also impact the air-sea heat fluxes. A recent study by Villas Bôas et al.^23^ found that mesoscale eddy features in the South Atlantic account for as much as 20% of the variance seen in the surface turbulent heat flux. Zhai and Greatbatch^24^ also found that the presence of eddies increased the surface heat flux in the northwest Atlantic.

The high correlation (r = 0.82 with p_value < 0.005) between the seasonal temperature difference and the ring formation difference (Fig. 3b) results from the WCR formations being highly correlated (r = 0.85 for both with p_value < 0.005) with the seasonal cycles of both Sargasso and slope (Fig. 3c). Figure 3b,c clearly show the in-phase (out-of-phase) seasonality of the Sargasso and slope thermostad (upper 50m) with WCR (CCR). The difference between the seasonal cycles of Sargasso and slope hints more towards the mechanism alluded by Gill^25^ in which the changes in the density structure of the upper ocean can lead to significant changes in the stability of the upper ocean, meaning that the ring production might be affected by local heating/cooling. This hypothesis was later observed to be working for the North Pacific Subtropical Countercurrent^26^. However, there are other mechanisms such as seasonal reduction in dissipation of EKE^27,28^, and seasonally varying Ekman pumping^29^ driving available potential energy to peak in winter and then followed by instability growth in spring leading to summer peaking of eddy formation. It is interesting to note that these hypotheses and ideas did not explicitly distinguish between Cold and Warm Core Rings from a meandering current.

The interannual asymmetry stems primarily from the observed regime shift of the WCR around 2000, and the absence of this shift in the CCR formation time-series over the last forty years. The increase in number of WCRs was attributed to increased instability in the Gulf Stream in recent years due to several factors such as (i) enhanced stratification^30^; (ii) changes in the internal dynamics of the Stream (transport, latitudinal movement, and subsequent interaction with DWBC and NESC)^31–34^ and (iii) changes in the large-scale atmospheric forcing (North Atlantic Oscillation, strength and shift of westerlies)^35^ or a combination of these as discussed in Gangopadhyay et al.^4^. Note that large-scale atmospheric patterns such as the Pacific Decadal Oscillation (PDO) and the NAO were not correlated with the observed time-series presented in Fig. 2a. A recent study by Li et al.^36^ provided evidence of a substantial 5.3% increase of stratification ($N^2$, buoyancy frequency) over 2000 m and 6.9% in the upper 200 m over the period 1960–2018 globally. These authors noted a 4.1% increase of stratification for the whole Atlantic basin up to 2000 m, and the slope and shelf region in the Atlantic has seen an even more dramatic increase. For example, an almost doubling of stratification between 2003 and 2014 for shelf water was observed by Harden et al.^37^ over the New England shelf. The coastal ocean experiences advection from freshwater inputs, which is important to increasing stratification in addition to air-sea flux anomalies. Untangling the various effects responsible for the interannual and seasonal asymmetries observed in the Gulf Stream ring formation will require further targeted dynamical and modeling studies.

The local impact of increasing warm rings on the Slope might have contributed to the observed long-term changes in sea surface temperature^30^, recent warming of the New England shelf near the Pioneer Array region^38^, and more frequent marine heatwaves^39^. It is conceivable that the observed asymmetric changes in the Gulf Stream Ring formation would not only cause local changes in the slope and on the shelf, such an asymmetry could also affect the air-sea fluxes in the marine atmospheric boundary layer which in turn could change the overlying atmosphere^3^. The noted Regime Shift Asymmetry could be seen as a change in the natural state of the Gulf Stream (e.g., baroclinicity, transport, width and meandering path) and further research, such as idealized numerical process studies, need to be conducted to understand the dynamics of the causes and consequences of such a profound shift in the behavior of the Gulf Stream.

## Methods

Our main data are two censuses created from a set of charts prepared by one of the co-authors (Jenifer Clark) from 1980 through 2019^40^. Using satellite infra-red (IR) imagery, satellite altimetry data, and surface in-situ temperature data, oceanographic analyses were produced for this region in the form of composite charts at 2–3 day intervals in a consistent manner. Each of these charts was annotated with identifiable and tractable multiscale ocean features within the region 80–55$^{\circ }$ W and 25–45$^{\circ }$ N (Gulf Stream, Warm Core Ring, Cold Core Ring, Shelf-Slope front, other eddies and finer-scale features). An example chart can be seen in Gangopadhyay et al.^4^. National Oceanic and Atmospheric Administration (NOAA) and the Bedford Institute of Oceanography (BIO) used these charts from 1980 to 2004 for extracting the Gulf Stream position and related Warm Core Ring locations and sizes. We reprocessed all the charts from 1980 to 2019 using a Geographical Information System (GIS) to establish a comprehensive, consistent and accurate database (see Methodology of Gangopadhyay et al.^4,5^ for details for the Warm Core Rings)^41–43^.

Following a similar methodology, a separate Cold Core Ring Census was developed by co-authors (Silver and Gangopadhyay). This robust census for Cold Core Ring formation, i.e. location and time, was developed for the full domain ($75^{\circ }$–$55^{\circ }\,\hbox {W}$) and for four zones (Zone1: $75^{\circ }$–$70^{\circ }\,\hbox {W}$; Zone 2: $70^{\circ }$–$65^{\circ }\,\hbox {W}$; Zone 3: $65^{\circ }$–$60^{\circ }\,\hbox {W}$ and Zone 4: $60^{\circ }$–$55^{\circ }\,\hbox {W}$) (see Fig. 1).

Following the methodology of Gangopadhyay^4^, a regime shift analysis was carried out on the interannual Cold Core Ring formations using three different methods. The first was Rodionov regime shift detection which can be run on either the data’s mean or variance, here it was used on the mean. This test uses Student’s t-test sequentially to determine if the current mean ($year_1$ through $year_n$) is significantly different from the new mean ($year_1$ through $year_{n+1}$). In this test one can also choose the cutoff length to start the sequence. Here we ran the test with cut off length of 5, 8, 10, 12, 14, 16, 18, and 20. For cutoff lengths greater then 12, no shift was detected for the Cold Core Rings. The Warm Core Ring data showed significant regime shifts for any cut-off length greater than 10.

The other supplementary methods used were the changepoint analysis in R^44^ and the changepoint detection in MATLAB^45–47^. Both methods gave no significant regime shifts for the Cold Core Rings.

To calculate the $\Delta T$, (difference between Sargasso and Slope temperatures) the monthly climatology of the Slope and Sargasso waters were collected. To do this, two polygons, one with the southwest corner at ($73.625^{\circ }\,\hbox {W}$, $33.875^{\circ }\,\hbox {N}$) to the northeast corner at ($58.125^{\circ }\,\hbox {W}$, $38.125^{\circ }\,\hbox {N}$) in the Sargasso and similarly another one from ($73.625^{\circ }\,\hbox {W}$, $37.375^{\circ }\,\hbox {N}$) to ($58.125^{\circ }\,\hbox {W}$, $41.625^{\circ }\,\hbox {N}$) in the Slope were considered as representative areas. Both polygons span regions parallel to the mean path of the Gulf Stream and are away from the Stream on either side to minimize the Stream’s meandering influence on the respective water mass compositions. The WOA18 $1/4^{\circ }$ resolution monthly climatology^21^ was used to compute the temperature difference over a vertical scale of 50 meters.

## Data Availability

The datasets generated during and/or analyzed during the current study are available from the corresponding author on reasonable request. The Warm Core Ring Census for 1980–2017 is available from Biological and Chemical Oceanography Data Management office (BCO-DMO), https://doi.org/10.26008/1912/bco-dmo.810182.1.
